# Insufficient Level of Physical Activity and Its Effect on Health Costs in Low- and Middle-Income Countries

**DOI:** 10.3389/fpubh.2022.937196

**Published:** 2022-06-27

**Authors:** Wei Liu, Abbas Dostdar-Rozbahani, Fahime Tadayon-Zadeh, Mohsen Akbarpour-Beni, Mohammad Pourkiani, Fatemeh Sadat-Razavi, Vahid Barfi, Valiollah Shahedi

**Affiliations:** ^1^Key Research Base of Humanities and Social Sciences in Colleges and Universities in Anhui Province–Quality Education Research Center for College Students of Anhui Xinhua University, Hefei, China; ^2^Department of Physical Education, Anhui Vocational and Technical College of Sports, Hefei, China; ^3^Department of Physical Education and Sport Sciences, Parand Branch, Islamic Azad University, Parand, Iran; ^4^Department of Exercise Physiology, Central Tehran Branch, Islamic Azad University, Tehran, Iran; ^5^Department of Sports Sciences, University of Qom, Qom, Iran; ^6^Department of Sport Management, Shahid Beheshti University, Tehran, Iran

**Keywords:** cost, diseases, health, income, physical activity, WHO

## Abstract

Evidence suggests that mortality attributed to noncommunicable diseases (NCDs) will increase from 38 million in 2012 to 52 million by 2030. The recent epidemiological data show that these diseases are increasing in low- and middle-income countries so that about 80% of all deaths of NCDs occurred in low- and middle-income countries. It has been estimated that an insufficient level of physical activity leads to a large share of the burden of these diseases. Evidence suggests that the rate of insufficient levels of physical activity in low- and middle-income countries has increased over the past 15 years. The authorities and policymakers must be advocated with consistent evidence from low- and middle-income countries on productivity loss and increased healthcare costs due to the absence or insufficient levels of physical activity. It is also necessary to include physical activity across all policies to prevent possible escalation of the economic burden related to physical inactivity in the near future.

## Introduction

According to the WHO, any bodily movement produced by skeletal muscles that requires energy expenditure, including activities undertaken while playing, working, carrying out household chores, engaging in recreational pursuits, and traveling, is called physical activity (1).

The current physical activity recommendation is at least 150 min of moderate-intensity physical activity or 75 min of vigorous-intensity physical activity throughout the week for adults/elderly and 60 min of moderate-intensity to vigorous-intensity physical activity daily for children aged 5–17 years (2).

It is proven that regular physical activity has many physical and mental health benefits. For example, it is associated with reduced rates of cardiovascular disease, stroke, obesity, cancer, diabetes, as well as anxiety, depression, and other mental health problems (3).

Also, regular physical activity can lead to improvements in functional health, muscular and cardiorespiratory fitness, cognitive function, quality of life, and wellbeing (3).

As one of the lifestyle behaviors, physical activity reduces the risk of all-cause mortality by about 33% (4). However, the absence or insufficient level of PA causes many health problems. Annually, about 3.2 million deaths (including 9% premature deaths) are due to insufficient levels of physical activity. In 2010, it was also the cause of 69.3 million disability-adjusted life years (DALYs) worldwide (1).

Evidence suggests that mortality attributed to non-communicable diseases (NCDs) will increase from 38 million in 2012 to 52 million by 2030 (5).

It has been estimated that an insufficient level of physical activity leads to a large share of the burden of main NCDs (about 10% in breast and colon cancer burden, about 7% in type 2 diabetes burden, and about 6% in chronic heart disease burden) (1).

## Insufficient Levels of Physical Activity and Ncds in Low- and Middle-Income Countries

In the rich and developed countries, NCDs, such as diabetes, coronary artery disease, cancer, obesity, chronic respiratory diseases, and mental illnesses, such as depression, are until recently, defined as health problems associated with economic development (1).

However, recent epidemiological data show that these diseases are increasing in low- and middle-income countries so that the number of mortalities due to these diseases has increased. In general, about 60% of all deaths (equivalent to 35 million deaths) are due to NCD, of which 80% occurred in low- and middle-income countries, so about 50% occurred before the age of 70 years (6).

In 2020, NCDs accounted for 80% of the global burden of disease, which accounted for 7 out of every 10 deaths in low- and middle-income countries (7).

Some studies document the known risk factors for significant NCDs in low- and middle-income countries (8, 9). One of the critical risk factors is physical inactivity (10).

In 2016, the prevalence of insufficient levels of physical activity worldwide was about 27.5%. This prevalence was about 16.2 and 26% in low- and middle-income countries, respectively. In addition, the prevalence rate was higher among schoolgoing adolescents in these countries (11).

Evidence suggests that the rate of insufficient levels of physical activity in low- and middle-income countries has increased over the past 15 years (12).

Due to the prevalence of the COVID-19 pandemic, lifestyle has also been affected. Some studies show that physical inactivity has increased during the pandemic, which is a sign of deteriorating conditions in low- and middle-income countries.

## Income Inequality and Physical Activity

The models estimate that with a point increasing income inequality, insufficient physical activity for the whole population in high-income countries increases (2.2% for men and 3.7% for women) (11).

Guthold et al. (11) observed 5% increase in the absence or insufficient physical activity in high-income countries over 15 years. They estimated that the effect size of the income inequality was large. Also, this association was weaker by about a third in income countries (11).

It seems that income inequality might be a determinant of insufficient physical activity and poor health; therefore, it needs to be more clearly considered and taken into account.

In large areas like countries or states, negative correlations are usually found between income inequality and health outcomes, while findings are less consistent in small areas like cities (13). This shows that income inequality could act as a global scale at the societal level, and for this reason, high-level policy measures are required to address it.

As countries transition from low- and middle-income to high-income economies, reducing income inequality can increase physical activity equity and global physical activity and prevent raise in insufficient physical activity and activity gender gap.

Another problem with income inequality in high-income settings vs. low-income countries is differences in the nature of the physical activity and whether it is utilitarian or volitional physical activity.

A recent study indicated that recreational or leisure-time physical activity was the only consistent relationship between socioeconomic status and self-reported physical activity so that people with low socioeconomic status did not have resources to direct to recreational or leisure-time physical activity and were more actively engaged in other domains of physical activity (14).

A study in the African region, most of which were low- and middle-income countries, found that about 79% of people were meeting the physical activity recommendations of WHO (15). However, only 5.3% accounted for leisure time activities and the vast majority of physical activity was utilitarian, in the form of transport-related (46.3%) or occupational (48.6%) physical activity (15).

It seems that the broader upstream factors, such as access to facilities and resources, infrastructure, and social determinants, such as safety from crime, affect the relationship between income inequality and its effect on the choice of physical activity in high-income countries compared to low- and middle-income countries.

## Health Costs Due to Physical Inactivity in Low- and Middle-Income Countries

Most of the estimates of the economic costs of physical inactivity are limited to direct healthcare costs and almost all were conducted in high-income countries. Although an article in 2013 examined this issue globally, there is no evidence of the economic burden of physical inactivity in low-income countries in local studies. This point is the main limitation, because it is now estimated that most of the global NCD burden and levels of physical inactivity are in low- and middle-income countries (16).

However, direct healthcare costs due to physical inactivity in low- and middle-income countries were about INT$10.3 billion in 2013. This cost is about 19% of direct healthcare costs attributable to physical inactivity worldwide (13).

Also, the estimated direct health costs due to physical inactivity in China were USD$3.5 billion in 2007 (17). Global analysis in 2013 estimated that direct healthcare cost attributed to physical inactivity was INT$3.1 billion for China, INT$1.6 billion for Brazil, INT$540 million for Iran, INT$699 million for Mexico, and INT408 million for South Africa (13).

In addition to these direct costs, inpatient and outpatient costs due to physical inactivity in 2000 were about US$0.4 and US$0.9 billion, respectively, in China (18).

In China, about 15.2% of the total cost of main NCDs in 2007 was related to the cost of chronic diseases attributed to physical inactivity, whereas 15% of the cost of inpatients in the Brazilian Health Care System in 2013 was related to physical inactivity (13).

In addition to the direct effects on health costs, physical inactivity also has indirect effects. For example, deaths due to physical inactivity in low- and middle-income countries contributed to a productivity loss of about INT$5.3 billion. This amount covers about 39% of productivity loss due to deaths related to physical inactivity worldwide (13). In low- and middle-income countries, about 10.1 million DALYs were due to physical inactivity, which is 75% of all DALYs worldwide (13).

Also, the indirect cost of physical inactivity and the estimated indirect cost due to productivity loss by deaths related to physical inactivity in China was about USD$3.3 billion and INT$1.78 billion, respectively (17).

## Discussion

Non-communicable diseases including diabetes, cancer, and heart and respiratory diseases currently cause 7 out of every 10 deaths worldwide. Evidence suggests that 85% of premature deaths (between ages 30–69 years) from these diseases occur in low- and middle-income countries, making them a huge health burden. However, the impact of these diseases on low- and middle-income countries is often underestimated. According to a new WHO report, if low- and middle-income countries consider an additional investment of less than a dollar per person per year to prevent and treat NCDs, close to 7 million deaths could be prevented by 2030. With the right strategic investments and evidence-based policies, low- and middle-income countries can change their disease trajectory and deliver significant economic and health gains for their citizens (1).

The absence or insufficient level of physical activity is one of the risk factors for chronic diseases that increase health-related costs. The economic burden of insufficient levels of physical activity and the inverse association of the economic cost of chronic diseases with physical activity were revealed by many studies (19).

Some studies showed that physical activity level increases with the increase in Human Development Index value (20) and the decrease in the Gini index, which is an index of wealth distribution of populations and inequality of income (21). Although physical activity level alone has shown a negative association with income level in low- and middle-income countries, the quality and quantity of physical activity have shown a positive association with the socioeconomic status of population subgroups as a whole in high-income countries (13).

It is important to identify strategies for physical activity promotion and cost-effective interventions in LMICs, and they should be well considered. For example, prescribing regular walking alone or along with medication as a cost-effective intervention and treatment regime for many chronic diseases can be considered to be implemented in low- and middle-income countries, because regular walking has a positive association with less medical expenditure (21).

Other problems are insufficient health research capacity and limited capacity and funding to conduct health economic research in low- and middle-income countries. Due to limited patient information systems and the absence of routine cost accounting systems in low- and middle-income countries, there are challenges in conducting economic evaluations in this context (22).

In general, a larger proportion of disease burden due to physical inactivity is consistently highlighted as a problem in low- and middle-income countries.

Although it is predicted that the economic burden of physical inactivity in low- and middle-income countries will be escalated in the near future, the current estimates of the economic burden of physical inactivity are huge in comparison with the income level of these countries. For this reason, it is important to draw the attention of policymakers and authorities to physical activity promotion as a priority measure in low- and middle-income countries.

Given the problems raised, it is suggested that low-income countries use low-cost interventions, such as walking, to improve the health of their citizens. Also, schools, as one of the environments with which most children and adolescents interact, can be an excellent and low-cost way to promote health-oriented behaviors as well as physical literacy. Another suggestion is a system of referral of physical activity from health centers to cheap health centers in order to cover a large part of the society.

## Conclusion

The authorities and policymakers must be advocated with consistent evidence from low- and middle-income countries on productivity loss and increased healthcare costs due to the absence or insufficient levels of physical activity.

It is also necessary to include physical activity across all policies to prevent possible escalation of the economic burden related to physical inactivity in the near future.

## Author Contributions

MA-B and WL: designed the work, drafted the manuscript, and participated in writing the manuscript. FT-Z, MP, FS-R, and VB: gathered information and researched literature. AD-R and VS: revised the work. All authors read and approved the final manuscript.

## Conflict of Interest

The authors declare that the research was conducted in the absence of any commercial or financial relationships that could be construed as a potential conflict of interest.

## Publisher's Note

All claims expressed in this article are solely those of the authors and do not necessarily represent those of their affiliated organizations, or those of the publisher, the editors and the reviewers. Any product that may be evaluated in this article, or claim that may be made by its manufacturer, is not guaranteed or endorsed by the publisher.
